# Linking genome content to biofuel production yields: a meta-analysis of major catabolic pathways among select H_2_ and ethanol-producing bacteria

**DOI:** 10.1186/1471-2180-12-295

**Published:** 2012-12-18

**Authors:** Carlo R Carere, Thomas Rydzak, Tobin J Verbeke, Nazim Cicek, David B Levin, Richard Sparling

**Affiliations:** 1 Department of Biosystems Engineering, University of Manitoba, Winnipeg, MB, Canada, R3T 5V6; 2 Department of Microbiology, University of Manitoba, Winnipeg, MB, Canada, R3T 5V6

## Abstract

**Background:**

Fermentative bacteria offer the potential to convert lignocellulosic waste-streams into biofuels such as hydrogen (H_2_) and ethanol. Current fermentative H_2_ and ethanol yields, however, are below theoretical maxima, vary greatly among organisms, and depend on the extent of metabolic pathways utilized. For fermentative H_2_ and/or ethanol production to become practical, biofuel yields must be increased. We performed a comparative meta-analysis of (i) reported end-product yields, and (ii) genes encoding pyruvate metabolism and end-product synthesis pathways to identify suitable biomarkers for screening a microorganism’s potential of H_2_ and/or ethanol production, and to identify targets for metabolic engineering to improve biofuel yields. Our interest in H_2_ and/or ethanol optimization restricted our meta-analysis to organisms with sequenced genomes and limited branched end-product pathways. These included members of the Firmicutes, Euryarchaeota, and Thermotogae.

**Results:**

Bioinformatic analysis revealed that the absence of genes encoding acetaldehyde dehydrogenase and bifunctional acetaldehyde/alcohol dehydrogenase (AdhE) in *Caldicellulosiruptor*, *Thermococcus*, *Pyrococcus*, and *Thermotoga* species coincide with high H_2_ yields and low ethanol production. Organisms containing genes (or activities) for both ethanol and H_2_ synthesis pathways (i.e. *Caldanaerobacter subterraneus* subsp*. tengcongensis*, *Ethanoligenens harbinense*, and *Clostridium* species) had relatively uniform mixed product patterns. The absence of hydrogenases in *Geobacillus* and *Bacillus* species did not confer high ethanol production, but rather high lactate production. Only *Thermoanaerobacter pseudethanolicus* produced relatively high ethanol and low H_2_ yields. This may be attributed to the presence of genes encoding proteins that promote NADH production. Lactate dehydrogenase and pyruvate:formate lyase are not conducive for ethanol and/or H_2_ production. While the type(s) of encoded hydrogenases appear to have little impact on H_2_ production in organisms that do not encode ethanol producing pathways, they do influence reduced end-product yields in those that do**.**

**Conclusions:**

Here we show that composition of genes encoding pathways involved in pyruvate catabolism and end-product synthesis pathways can be used to approximate potential end-product distribution patterns. We have identified a number of genetic biomarkers for streamlining ethanol and H_2_ producing capabilities. By linking genome content, reaction thermodynamics, and end-product yields, we offer potential targets for optimization of either ethanol or H_2_ yields through metabolic engineering.

## Background

Fuel derived from waste-stream lignocellulosic biomass via consolidated bioprocessing is a renewable and carbon-neutral alternative to current petroleum-based fuels [1-3]. Consequently, considerable effort is being made to characterize species capable of efficiently converting lignocellulosic substrates into biofuels. An ideal biofuel producing microorganism should posses several key features, including: (i) high yields of the desired product, (ii) simultaneous utilization of sugars (cellulose, hemicellulose, pectin), and (iii) growth at elevated temperatures, and (iv) low product inhibition. Recent studies have focused on the characterization of numerous cellulose and hemicellulose degrading species of bacteria [4-6]. To fully exploit the biofuel producing potential of these organisms, several genomes have been sequenced and are now available for analysis (http://genome.jgi-psf.org/). While some hemicellulolytic or cellulolytic microorganisms are capable of hydrogen (H_2_) or ethanol production via fermentation, end-product yields typically are far lower than their maximum theoretical values (4 mol H_2_ or 2 mol ethanol per mol glucose) when cells are grown in pure culture. This is due to the presence of branched catabolic pathways that divert carbon and/or electrons away from a particular desired end-product [7]. Strategies that optimize yields for a single biofuel (H_2_ or ethanol) can only be developed through a detailed knowledge of the relationships between genome content, gene and gene product expression, pathway utilization, and end-product synthesis patterns.

Given that our primary focus is to optimize H_2_ and/or ethanol yields, we restricted our meta-analysis to sequenced organisms with limited branched end-product pathways (i.e. organisms that do not produce butyrate, butanol, propionate, propanol, and acetoin) for which end-product data was available. These included members of the Firmicutes (*Clostridium*, *Caldicellulosiruptor*, *Thermoanaerobacter*, *Caldanaerobacter*, *Ethanoligenens*, *Geobacillus*, and *Bacillus* species), Euryarchaeota (*Thermococcus* and *Pyrococcus* species), and Thermotogae (*Thermotoga* species). A list of species analyzed and corresponding GenBank accession numbers are summarized in Table 1. With the exception of *Caldanaerobacter subterraneus* subsp*. tengcongensis*, *Thermoanaerobacter pseudethanolicus*, *Pyrococcus furiosus*, *Geobacillus thermoglucosidasius*, and *Bacillus cereus*, all organisms were capable of cellulose and/or xylan saccharification.

**Table 1:** **H**_**2**_**and ethanol producing organisms included in meta-analysis of end-product yields and genome content**

**Organism**	**Synonyms**	**Taxon ID**	**GenBank #**	**Sequencing Center**	**Phyla**	**C sources**
*Caldicellulosiruptor saccharolyticus* DSM 8903		351627	NC_009437	DOE Joint Genome Institute	F	S,C,X
*Caldicellulosiruptor besci* DSM 6725	*Anaerocellum thermophilum*; Z-1320	521460	NC_012036	DOE Joint Genome Institute	F	S,C,X
*Pyrococcus furiosus* DSM 3638		186497	AE009950	Univ of Maryland, Univ of Utah	E	S,C,X
*Thermococcus kodakaraensis* KOD1		69014	NC_006624	Kwansei Gakuin Univ, Kyoto University	E	S
*Thermotoga neapolitana* DSM 4359	ATCC 49049; JCM 10099; NS-E	309803	NC_011978	Genotech corp.	T	S,C
*Thermotoga petrophila* RKU-1		390874	NC_009486	DOE Joint Genome Institute	T	S,C,X
*Thermotoga maritima* MSB8	DSM 3109	243274	NC_000853	J. Craig Venter Institute	T	S,C,X
*Caldanaerobacter subterraneus* subsp*. tengcongensis* MB4	*Thermoanaerobacter tencongensis*	273068	NC_003869	Beijing Genomics Institute, The Institute of Microbiology, China	F	S
*Ethanoligenens harbinense* YUAN-3 T	DSM 18485	663278	NC_014828	DOE Joint Genome Institute	F	S,C
*Clostridium cellulolyticum* H10		394503	NC_011898	DOE Joint Genome Institute	F	S,C,X
*Clostridium phytofermentans* ISDg	ATCC 700394	357809	NC_010001	DOE Joint Genome Institute	F	S,C,X
*Clostridium thermocellum* ATCC 27405	DSM 1237	203119	NC_009012	DOE Joint Genome Institute, University of Rochester	F	S,C,X
*Clostridium thermocellum* DSM 4150	JW20	492476	ABVG00000000	DOE Joint Genome Institute	F	S,C,X
*Thermoanaerobacter pseudethanolicus* 39E	ATCC 33223	340099	NC_010321	DOE Joint Genome Institute	F	S,X
*Geobacillus thermoglucosidasius* C56-YS93		634956	NC_015660	DOE Joint Genome Institute	F	S
*Bacillus cereus* ATCC 14579	DSM 31	226900	NC_004721	Integrated Genomics Inc.	F	S

National Center for Biotechnology Information taxon IDs, GenBank accession numbers, corresponding sequencing centers responsible for the generation of the genome sequences data analyzed in this study are provided. Phyla (F; Firmicutes: E;Euryarchaeota: T; Thermotogae), and polymeric carbon sources degraded (S; starch: C; cellulose: X; xylose) by each organism are indicated).

We focused on the various metabolic branches involved in pyruvate formation from phosphoenolpyruvate (PEP) and subsequent catabolism of pyruvate into end-products. Although studies comparing the H_2_ and ethanol-producing potential of several cellulose degrading bacteria have been previously published [8-10], a comprehensive comparison of the major biofuel producing pathways at the genome level has not yet been reported. Here we present a comparison of the genes encoding proteins involved in (i) pyruvate metabolism, (ii) ethanol synthesis, and (iii) H_2_ metabolism, in order to rationalize reported end-product yields. Results indicate that the presence or absence of specific genes dictating carbon and electron flow towards end-products may be used to infer end-product synthesis patterns and help develop informed metabolic engineering strategies for optimization of H_2_ and ethanol yields. Furthermore, certain genes may be suitable biomarkers for screening novel microorganisms’ capability of producing optimal H_2_ or ethanol yields, and may be suitable targets for metabolic engineering strategies for optimization of either ethanol or H_2_ yields

## Methods

### Comparative analysis of genome annotations

All sequence data and gene annotations were accessed using the Joint Genome Institute’s Integrated Microbial Genomes (IMG) database [11]. Gene annotations presented in this paper reflect the numbering of the final assembly or most recent drafts available (July, 2012). Comparative analyses were performed using the IMG database. In brief, analyses of all genomes (Table 1) were conducted using three annotation databases independently: i) Clusters of Orthologs Groups (COGs) [12], ii) KEGG Orthology assignments (KO) [13], and (iii) TIGRFAMs [14]. Genes identified using a single database were cross-referenced against the others to identify genes of interest. Functional annotations of the identified genes were evaluated on a case-by-case basis and decisions regarding the annotation accuracy were made using a combination of manual analysis of genomic context, literature searches, and functional prediction through RPS-BLAST using the Conserved Domain Database website [15].

Hydrogenases were classified based on phylogenetic relationships of hydrogenase large subunits according to Calusinska *et al*. [16]. The evolutionary history was inferred using the Neighbor-Joining method [17]. The bootstrap consensus tree inferred from 1000 replicates is taken to represent the evolutionary history of the taxa analyzed [18]. The evolutionary distances were computed using the Poisson correction method [19] and are in the units of the number of amino acid substitutions per site. The analysis involved 50 amino acid sequences. All ambiguous positions were removed for each sequence pair. There were a total of 863 positions in the final dataset. Evolutionary analyses were conducted in MEGA5 [20]. Thermodynamic calculations were performed using values provided by Thauer *et al.*[21] and the CRC Handbook of Chemistry and Physics [21,22]. BioEdit v.7.0.9.0 [23] was used to perform sequence alignments.

## Results and discussion

### Survey of End-product yields

A literature survey of end-product yields (normalized to mol end-product per mol hexose equivalent) of the species surveyed in this study is summarized in Table 2. While it is difficult to perform a direct comparison of end-product yields from available literature due to different growth conditions employed (ex. growth substrate, carbon loading, reactor conditions, etc.), and further difficult to validate these data due to incomplete end-product quantifications and lack of corresponding carbon balances and oxidation/reduction (O/R) ratios, it still provides a good approximation of molar end-product yields based on substrate utilization. Calculated end-product yields reveal that the *Caldicellulosiruptor*, *Pyrococcus*, *Thermococcus*, and *Thermotoga* species surveyed, produced, in most cases, near-maximal H_2_ yields with concomitant CO_2_ and acetate production, and little or no ethanol, formate, and lactate [24-40]. It is important to note that while some studies [29-31,34,35,39] report lower overall end-product yields, likely due to a large amount of carbon flux being directed towards biomass production under a given growth condition, H_2_:ethanol ratios remain high. *Cal. subterraneus* subsp*. tengcongensis*, *E. harbinense*, and *Clostridium* species displayed mixed end-product fermentation patterns, with comparatively lower H_2_, CO_2_, and acetate yields, higher ethanol yields, and generally low formate and lactate yields [10,41-47]. *Ta. pseudethanolicus* produced the highest ethanol yields of the organisms surveyed with little concomitant H_2_, acetate, and lactate production, and no formate synthesis [48-50]. *G. thermoglucosidasius* and *B. cereus* produced the highest lactate and formate yields, moderate ethanol and acetate yields, and low H_2_ and CO_2_ yields [51,52].

**Table 2:** **Summary of end-product yields, optimal growth temperatures, total molar reduction values of H**_**2**_ **+ ethanol (*RV***_***EP***_**), and growth conditions employed**

**Organism**	**Growth temp (°C)**	**End products (mol/mol hexose equivalent)**							**Growth condition**	**Ref**
		**H**_**2**_	**CO**_**2**_	**Acetate**	**Ethanol**	**Formate**	**Lactate**	***RV***_***EP***_		
*Ca. saccharolyticus* DSM 8903	70	4.0	1.8	NR	ND	ND	ND	4.0	Cont., 1.1 g l^-1^ glucose (D = 0.09 h^-1^)	[24]
		3.6	1.5	1.6	ND	ND	ND	3.6	Cont., 4.1 g l^-1^ glucose (D = 0.1 h^-1^)	[24]
		3.5	NR	2.1	NR	NR	NR	3.5	Batch, 10 g l^-1^ sucrose	[25]
		2.5	1.4	1.4	ND	ND	0.1	2.5	Batch, 10 g l^-1^ glucose	[26]
*Ca. bescii* DSM 6725	75	✓	✓	✓	NR	NR	✓	NA		[27,28]
*P. furiosus* DSM 3638	90	3.8	1.9	1.5	0.1	NR	NR	4.0	Cont, cellobiose (D = 0.45 h^-1^)	[29]^A^
		3.5	1.0	1.4	ND	NR	ND	3.5	Batch, 1.9 g l^-1^, maltose	[30]^A^
		2.9	1.9	0.8	0.1	NR	ND	3.1	Batch, 2 g l^-1^ maltose	[31]^B^
		2.8	0.9	1.2	ND	NR	ND	2.8	Batch, 3.5 g l^-1^, cellobiose	[30]^A^
		2.6	1.4	1.0	ND	NR	NR	2.6	Cont, maltose (D = 0.45 h^-1^)	[29]^A^
*Th. kodakaraensis* KOD1	85	3.3	1.8	1.1	NR	NR	NR	3.3	Cont, starch (D = 0.2 h^-1^)	[32]^C^
*T. neapolitana* DSM 4359	80-85	3.8	2.0	1.8	ND	NR	0.1	3.8	Batch, 2.5 g l^-1^ glucose	[33]
		3.2	NR	1.9	NR	NR	NR	3.2	Batch (N_2_ sparged), 7.0 g l^-1^ glucose	[34]
		2.4	NR	1.1	NR	NR	0.7	2.4	Batch, 1.1 g l^-1^ glucose	[35]
		1.8	NR	1.0	NR	NR	NR	1.8	Batch, 7.5 g l^-1^ glucose	[40]
		1.8	NR	1.5	NR	NR	NR	1.8	Batch, 7.0 g l^-1^ glucose	[34]
*T. petrophila* RKU-1	80	3.7	0.4	1.8	NR	NR	0.3	3.7	Batch, 1 g l^-1^ glucose	[36]
*T. maritima* MSB8	80	4.0	2.0	2.0	NR	ND	NR	4.0	Batch, 2 g l^-1^ glucose	[38]
		2.2	1.1	1.0	ND	NR	0.3	2.2	Batch, 3 g l^-1^ glucose	[39]
		1.7	NR	1.0	NR	NR	NR	1.7	Batch, 7.5 g l^-1^ glucose	[40]
*Cal. subterraneus* subsp*. tengcongensis* MB4	75	2.8	NR	1.4	0.6	NR	ND	4.0	Cont, starch (D = 0.27 h^-1^)	[42]
		NR	NR	2.0	ND	NR	ND	NA	Cont (N_2_ sparged), glucose (D = 0.24 h^-1^)	[42]
		0.3	1.5	1.0	0.7	NR	ND	1.7	Batch, 4 g l^-1^ glucose	[41]
*E. harbinense* YUAN-3 T	35	2.8	✓	0.7	1.1	ND	ND	5.0	Batch, 20 g l^-1^ glucose	[43]
*C. cellulolyticum* H10	37	1.6	1.0	0.8	0.3	ND	NR	2.2	Batch, 5 g l^-1^ cellulose	[44]
		1.8	1.1	0.8	0.4	ND	NR	2,6	Batch, 5 g l^-1^ cellobiose	[44]
*C. phytofermentans* ISDg	35-37	Major	Major	0.6	1.4	0.1	0.3	NA	Batch, 34 g l^-1^ cellobiose	[45]
		1.0	0.9	0.6	0.5	0.1	NR	2.0	Batch, 5 g l^-1^ cellulose	[44]
		1.6	1.2	0.6	0.6	ND	NR	2.8	Batch, 5 g l^-1^ cellobiose	[44]
*C. thermocellum* ATCC 27405	60	0.8	1.1	0.7	0.8	0.3	ND	2.4	Batch, 1.1 g l^-1^ cellobiose	[10]
		1.0	0.8	0.8	0.6	0.4	0.4	2.2	Batch, 4.5 g l^-1^ cellobiose	[46]
*C. thermocellum* DSM 4150	60	1.8	1.7	0.9	0.8	ND	0.1	3.4	Batch, 2 g l^-1^ glucose	[47]
		0.6	1.8	0.3	1.4	ND	0.2	3.4	Batch, 27 g l^-1^ cellobiose	[47]
*Ta. pseudethanolicus* 39E	65	0.1	2.0	0.1	1.8	NR	0.1	3.7	Batch, 8 g l^-1^ glucose	[50]
		NR	NR	NR	1.6	NR	<0.1	3.2	1 g l^-1^ xylose	[48]
		NR	NR	0.4	1.0	NR	<0.1	2.0	Batch, 20 g l^-1^ xylose	[49]
		NR	NR	0.2	0.4	NR	1.1	0.8	Batch, 20 g l^-1^ glucose	[49]
*G. thermoglucosidasius* M10EXG^D^	60	NR	NR	0.6	0.4	1.0	0.9	0.8	Batch, 10 g l^-1^ glucose	[52]
*B cereus* ATCC 14579	35	NR	0.1	0.2	0.2	0.3	1.1	0.4	Batch, 3.6 g l^-1^ glucose	[51]

^A^ ~ 0.5 mol alanine per mol-hexose produced on cellobiose and maltose.

^B^Produces H_2_, CO_2_, volatile fatty acids, and NH_3_ on peptides in the absence of carbon source.

^C^ ~ 0.5 mol alanine per mol-hexose produced on starch.

^D^Only *G. thermoglucosidasuis* strain C56-TS93 has been sequenced but no end-product data is available. Strain M10EXG was used for end-product yield comparisons instead.

Abbreviations: NR, not reported; ND, not detected; NA, not applicable; Major, reported as major product without absolute values; ✓, reported as present with no values indicated; Cont, continuous culture; D, dilution rate.

While reported yields vary considerably for each organisms, it is important to note that different growth conditions may influence end-product yields through regulation of gene and gene product expression [42,53], and modulation of metabolic flux and intracellular metabolite levels [54,55] that may act as allosteric regulators [56,57]. Variations in fermentation conditions including substrate availability/dilution rates [46,53-55,58-61], substrate composition [54,62-67], media composition [55], pH [68], gas partial pressures [34,42,69,70], growth phase [57], and accumulation of end-products [47,62,69,71,72] have been shown to influence end-product yields. Hence, while genome content alone cannot be used to predict end-product yields with accuracy, it can reflect end-product distribution profiles.

### Genome comparison of pyruvate metabolism and end-product synthesis pathways

**Figure 1:**
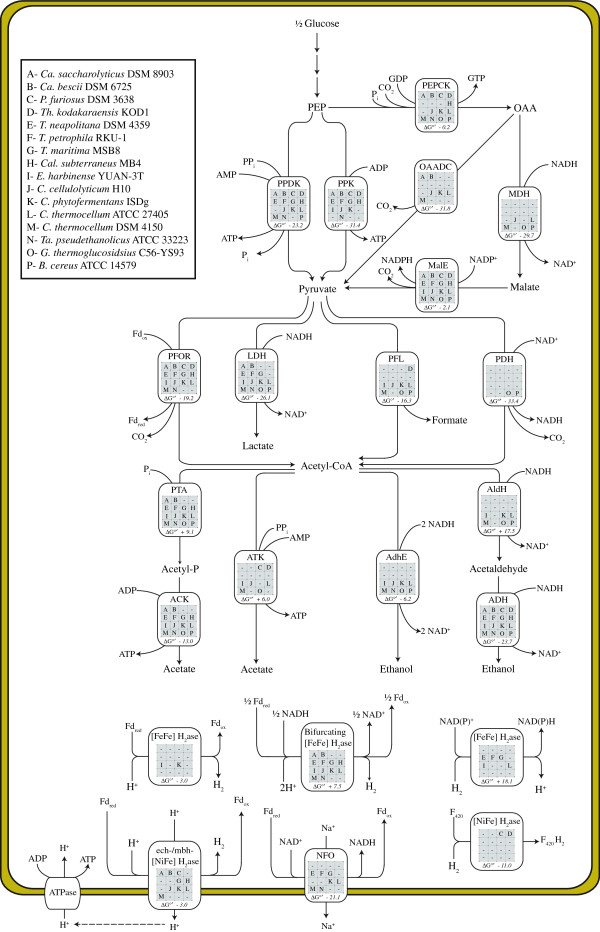
**Comparison of putative gene products involved in pyruvate metabolism and end-product synthesis among select hydrogen and ethanol-producing species.** Presence of putative gene products are indicated in matrix with respective letters corresponding to selected organism (see legend). Numbers indicate standard free energies of reaction (△G°’) corresponding to a particular enzyme. Abbreviations: PEPCK, phosphoenolpyruvate carboxykinase; OAADC, oxaloacetate decarboxylase; MDH, malate dehydrogenase; MalE, malic enzyme; PPK, pyruvate kinase; PPDK, pyruvate phosphate dikinase; LDH, lactate dehydrogenase; PFL, pyruvate formate lyase; PFOR, pyruvate:ferredoxin oxidoreductase; PDH, pyruvate dehydrogenase; ADH, alcohol dehydrogenase; ALDH, acetaldehyde dehydrogenase; AdhE, bifinctional acetaldehyde/alcohol dehydrogenase; ACK, acetate kinase; PTA, phosphotransacetylase; NFO, NADH:Fd oxidoreductase.

The assemblage of genes encoding proteins involved in pyruvate metabolism and end-product synthesis dictate, in part, how carbon and electron flux is distributed between the catabolic, anabolic, and energy producing pathways of the cell. The flow of carbon and electrons from PEP towards end-products may be separated into branch-points or nodes which include (i) the PEP/oxaloacetate/pyruvate node, (ii) the pyruvate/lactate/acetyl-CoA node, (iii) the acetyl-CoA/acetate/ethanol node, and the (iv) ferredoxin/NAD(P)H/H_2_ node [73]. Several different enzymes may be involved in the conversion of intermediate metabolites within these nodes. These enzymes, and the presence of corresponding genes encoding these proteins in each of the organisms surveyed, are summarized in Figure 1. The oxidation of electron carriers (NADH and/or reduced ferredoxin) is required for maintaining glycolytic flux and leads to the ultimate production of reduced products (ethanol, lactate, and H_2_). Thus, distribution of carbon and electron flux among different pathways can influence levels of reduced electron carrier pools, which in turn can dictate end-product distribution patterns. Genome content can be used to resolve the relationship between carbon and electron flux with end-product distribution.

#### Genes involved in pyruvate synthesis

All organisms considered in this study utilize the Embden-Meyerhof-Parnas pathway for conversion of glucose to PEP with the following notable variations. Alignments of key residues of phosphofructokinase (PFK) according to Bapteste *et al.*[74,75], suggest that *P. furiosus*, *Th. kodakaraensis*, *Cal. subterraneus* subsp*. tengcongensis*, *E. harbinense*, *G. thermoglucosidasius*, and *B. cereus* encode an ATP-dependent PFK, while *Thermotoga*, *Caldicellulosiruptor*, *Clostridium*, and *Thermoanaerobacter* species encode both an ATP-dependent PFK, as well as a pyrophosphate (PP_i_)-dependent PFK [74,75] (Additional file 1). Furthermore, while bacteria catalyze the oxidation of glyceraldehyde-3-P to 3-phosphoglycerate (yielding NADH and ATP) with glyceraldehydes-3-phosphate dehydrogenase (GAPDH) and phosphoglycerate kinase (PGK), archea (*P. furiosus* and *Th. kodakaraensis*) preferentially catalyze the same reaction via glyceraldehyde-3-phosphate ferredoxin oxidoreductase (GAPFOR). This enzyme reduces ferredoxin (Fd) rather than NAD^+^ and does not produce ATP [76].

In contrast to the generally conserved gene content required for the production of PEP, a number of enzymes may catalyze the conversion of PEP to pyruvate [73] (Figure 1; Table 3). PEP can be directly converted into pyruvate via an ATP-dependent pyruvate kinase (PPK), or via an AMP-dependent pyruvate phosphate dikinase (PPDK). All strains considered in this review encode both *ppk* and *ppdk*, with the exception of *C. thermocellum* strains, which do not encode a *ppk*, and *E. harbinense*, *G. thermoglucosidasius*, and *B. cereus*, which do not encode *ppdk*. Given that the formation of ATP from ADP and P_i_ is more thermodynamically favorable than from AMP and PP_i_ (△G°’ = 31.7 vs. 41.7 kJ mol^-1^), production of pyruvate via PPK is more favorable than via PPDK [21].

**Table 3:** Genes encoding proteins involved in interconversion of phosphenolpyruvate and pyruvate

**Organism**	**Gene**						
	***eno***	***ppk***	***ppdk***	***pepck***	***oaadc***	***mdh***	***malE***
Standard free energy (ΔG°’)	ND	−31.4	−23.2	−0.2	−31.8	−29.7	−2.1
*Ca. saccharolyticus* DSM 8903	Athe_1403	Athe_1266	Athe_1409	Athe_0393	Athe_1316-1319		Athe_1062
*Ca. bescii* DSM 6725	Csac_1950	Csac_1831	Csac_1955	Csac_0274	Csac_2482-2485		Csac_2059
*P. furiosus* DSM 3638	PF0215	PF1188	PF0043	PF0289			PF1026
	PF1641						
*Th. kodakaraensis* KOD1	TK1497	TK0511	TK0200	TK1405			TK1963
	TK2106		TK1292				
*T. neapolitana* DSM 4359	CTN_1698	CTN_0477	CTN_0413				CTN_0126
*T. petrophila RKU-1*	Tpet_0050	Tpet_0716	Tpet_0652				Tpet_0379
*T. maritima* MSB8	TM0877	TM0208	TM0272				TM0542
*Cal. subterraneus* subsp*. tengcongensis* MB4^A^	TTE1759	TTE1815	TTE0164	TTE1783			TTE2332
			TTE0981				
*E. harbinense* YUAN-3 T	Ethha_2662	Ethha_0305					Ethha_0739
*C. cellulolyticum* H10	Ccel_2254	Ccel_2569	Ccel_2388	Ccel_0212	Ccel_1736-1738	Ccel_0137	Ccel_0138
*C. phytofermentans* ISDg	Cphy_3001	Cphy_0741	Cphy_0651	Cphy_3853	Cphy_2433-2434		Cphy_0409
		Cphy_2900					
*C. thermocellum* ATCC 27405	Cthe_0143		Cthe_1253	Cthe_2874	Cthe_0699-0701	Cthe_0345	Cthe_0344
			Cthe_1308				
*C. thermocellum* DSM 4150			CtherDRAFT_1661	CtherDRAFT_1742	CtherDRAFT_0819-0822	Yes^A^	Yes^A^
			CtherDRAFT_1896				
*Ta. pseudethanolicus* 39E	Teth39_0735	Teth39_0684	Teth39_1358	Teth39_0711			Teth39_0337
			Teth39_2098				
*G. thermoglucosidasius* C56-YS93	Geoth_0446	Geoth_0898		Geoth_0811		Geoth_0904	Geoth_1713
						Geoth_3508	Geoth_2444
*B.cereus* ATCC 14579	BC5135	BC3323	BC3087	BC4762		BC4592	BC0580 NAD)
		BC4599				BC2959	BC1741 (NAD)
							BC4604 (NADP)

^A^Genes have been verified by PCR amplification (*unpublished*).

Abbreviations: *eno*, enolase; *ppk*, pyruvate kinase; *ppdk*, pyruvate phosphate dikinase; *pepck*, phosphoenolpyruvate carboxykinase; *oaadc*, oxaloacetate decarboxylase; *mdh*, malate dehydrogenase; *malE*, malic enzyme.

Flux balance analysis integrated with RNAseq data suggests higher carbon and electron flux in *C. thermocellum* ATCC 27405 is directed through enzymes capable of direct, rather than indirect, conversion of PEP to pyruvate [77]. However, *C. cellulolyticum* mutation studies suggests that a portion of PEP can also be converted to pyruvate via the “malate shunt” [78]. This PPK/PPDK bypass system utilizes either (i) phosphoenolpyruvate carboxykinase (PEPCK), malate dehydrogenase (MDH), and malic enzyme (MalE), or (ii) PEPCK and oxaloacetate decarboxylase (OAADC), for the interconversion of PEP and pyruvate (Figure 1). While PEPCK provides a pathway for energy conservation via ATP (or GTP) production, MDH and MalE permit transhydrogenation from NADH to NADP^+^[71], generating additional reducing equivalents required for biosynthesis. *G. thermoglucosidasius*, *B. cereus*, *C. thermocellum* (ATCC 27405), and *C. cellulolyticum* contain *pepck*, *mdh* and *malE* suggesting that they are capable of transhydrogenation using these proteins. Although the draft genome of *C. thermocellum* DSM 4150 does not include genes encoding MDH and MalE, we have verified their presence via PCR amplification (unpublished results). Deletion of *mdh* in *C. cellulolyticum* resulted in significant increases in lactate, and to a lesser extent ethanol yields, and reduced acetate production when grown on cellulose demonstrating carbon and electron flux through MDH in wild type strains [78]. It seems evident that in the absence of MDH, transhydrogenation was reduced, and thus the resulting increase in NADH:NADPH ratios promote lactate and ethanol production, while decreasing NADPH levels for biosynthesis.

A number of organisms analyzed encode *pepck* and *oaadc* (*Ca. bescii*, *Ca. saccharolyticus*, *C. cellulolyticum*, *C. phytofermentans*, and *C. thermocellum*), also allowing for indirect conversion of PEP to pyruvate via an oxaloacetate intermediate. While the redirection of carbon and electron flux through this pathway likely has little effect on product yields, synthesis of GTP, versus ATP, may promote transcription and protein synthesis. Finally, *Cal. subterraneus*, *E. harbinense*, *P. furiosus*, *Th. kodakaraensis*, *Ta. pseudethanolicus*, and *Thermotoga* species do not encode all of the proteins required for a “malate shunt” and consequentially the catalysis of PEP to pyruvate must be achieved via PPK and/or PPDK.

#### Genes involved in pyruvate catabolism

The pyruvate/lactate/acetyl-CoA node plays an important role in regulating carbon flux and electron distribution and dramatically affects end-product distribution. The NADH-dependent reduction of pyruvate to lactate via fructose-1,6-bisphosphate activated lactate dehydrogenase (LDH) [56] diverts reducing equivalents away from biofuels such as H_2_ and ethanol. Alternatively, the oxidative decarboxylation of pyruvate to acetyl-CoA via pyruvate dehydrogenase (*pdh*) or pyruvate:ferreodoxin oxidoreductase (*pfor*) generate NADH and reduced Fd, respectively. These reducing equivalents may then be oxidized during the production of H_2_ or ethanol (Figure 1). Pyruvate may also be catabolised to acetyl-CoA via pyruvate:formate lyase (*pfl*) yielding formate in the process. In some enterobacteria, formate is further oxidized to CO_2_, releasing H_2_, through the action of a multisubunit formate hydrogen lyase (FHL) complex [79]. However, *pfl* was not encoded in any of the organisms analysed.

With the exception of *Cal. subterraneus* subsp*. tengcongensis*, *P. furiosus*, and *Th. kodakaraensis*, *ldh* genes were identified in all organisms studied (Table 4). Surprisingly, while the production of lactate from pyruvate is highly favorable thermodynamically (△G°’ = − 26.1 kJ mol^-1-^), only *B. cereus*, *G. thermoglucosidasius*, and, under some conditions, *Ta. pseudethanolicus* and *T. neapolitana* produce high yields of lactate (> 0.5 mol mol-glucose^-1^). In all other organisms surveyed lactate production was either a minor end-product, not detected, or not reported under the reported growth conditions (Table 2). This suggests that the presence of *ldh* cannot be used to predict lactate production.

**Table 4:** Genes encoding proteins directly involved in pyruvate catabolism

**Organism**	**Gene**			
	***ldh***	***pdh***	***pfor***	***pfl***
Standard free energy (G°’)	−26.1	−33.4	−19.2	−16.3
*Ca. saccharolyticus* DSM 8903	Csac_1027		Csac_1458-1461	
			Csac_2248-2249	
*Ca. bescii* DSM 6725	Athe_1918		Athe_0874-0877	
			Athe_1708-1709	
*P. furiosus* DSM 3638			PF0965-PF0967, PF0971	
*Th. kodakaraensis* KOD1			TK1978, TK1982-1984	TK0289
*T. neapolitana* DSM 4359	CTN_0802		CTN_0680-CTN_0683	
*T. petrophila* RKU-1	Tpet_0930		Tpet_0905-Tpet_0908	
*T. maritima* MSB8	TM1867		TM0015-TM0018	
*Cal. subterraneus* subsp. *tengcongensis* MB4			TTE0445	
			TTE0960	
*E. harbinense* YUAN-3 T	Ethha_1350		Ethha_0231-0234	Ethha_1657
	Ethha_2705			
*C. cellulolyticum* H10	Ccel_2485		Ccel_0016	Ccel_2224
			Ccel_1164	Ccel_2582
*C. phytofermentans* ISDg	Cphy_1117 Cphy_1232		Cphy_0603 Cphy_3558	Cphy_1174
				Cphy_1417
				Cphy_2823
*C. thermocellum* ATCC 27405	Cthe_1053		Cthe_2390-2393	Cthe_0505
			Cthe_2794-2797	
			Cthe_3120	
*C. thermocellum* DSM 4150	CtherDRAFT_2943		CtherDRAFT_0414-0417	CtherDRAFT_2234
			CtherDRAFT_1182-1185	
			CtherDRAFT_1311	
*Ta. pseudethanolicus* 39E	Teth39_1997		Teth39_0289	
			Teth39_1842	
				
*G. thermoglucosidasius* C56-YS93	Geoth_3351	Geoth_0237-0239		Geoth_3895
		Geoth_1595-1597		
		Geoth_2366-2368		
		Geoth_2479-2480		
		Geoth_2860-2863		
*B.cereus* ATCC 14579	BC1924	BC3970-3973		BC0491
	BC4870			
	BC4996			

Abbreviations: *ldh*, lactate dehydrogenase; *pdh*, pyruvate dehydrogenase; *pfor*, pyruvate:ferredoxin oxidoreductase; *pfl*, pyruvate formate lyase.

LDH is, in fact, allosterically activated by fructose-1,6-bisphosphate in *C. thermocellum* ATCC 27405, *Ca. saccharolyticus*, and *Thermoanaerobacter brockii*[56,57,62,80]. While enzyme assays reveal high LDH activity in *C. thermocellum*[10,72], most studies report only trace amounts of lactate. Islam *et al*. [46], however, demonstrated that lactate production was triggered in stationary-phase batch cultures only under excess cellobiose conditions. In *Thermoanaerobacter brockii,* Ben-Bassat *et al*. reported elevated lactate production as a consequence of accumulated intracellular fructose-1,6-bisphosphate (FDP) when cultures were grown on glucose compared to starch [62]. Finally, Willquist and van Niel [57] reported that LDH in *Ca. saccharolyticus* was activated by FDP and ATP, and inhibited by NAD^+^ and PP_i_. An increase in fructose-1,6-bisphosphate, NADH:NAD^+^ ratios, and ATP:PP_i_ ratios was observed during the transition from exponential to stationary phase in *Ca. saccharolyticus* cultures, and was accordingly accompanied by lactate production [57].

All organisms analyzed encode either *pdh* or *pfor*, but not both (Table 4). While *G. thermoglucosidasius* and *B. cereus* encode *pdh*, all other organisms analyzed encode *pfor*. Although *Caldicellulosiruptor*, *Clostridia*, and *Thermoanaerobacter* species studied appear to encode a putative *pdh*, there has been no enzymatic evidence to support the presence of PDH in these species. Thus far, only PFOR activity has been verified in *C. cellulolyticum*[58,60] and *C. thermocellum*[10,72]. The putative E1, E2, and E3 subunits of the *pdh* complex (Csac_0874-0872) in *Ca. saccharolyticus* were designated simply as a keto-acid dehydrogenase by van de Werken *et al*. [81]. Similarly, while genes encoding a putative *pdh* (Teth_0790-0793) are present in *Ta. pseudethanolicus*, genomic context strongly supports that this putative *pdh* is part of an acetoin dehydrogenase complex, despite the absence of reported acetoin production. In *Clostridia* species, putative *pdh’*s (Cthe_3449-3450, Cthe_1543) may actually encode 2-oxo acid dehydrogenase complexes, which share a common structure and homology to pyruvate dehydrogenase. These include 2-oxoglutarate dehydrogenase, branched-chain alpha-keto acid dehydrogenase, acetoin dehydrogenase complex, and the glycine cleavage complex. All organisms that encode a *pfor* also encode a Fd-dependent hydrogenase (H_2_ase), bifurcating H_2_ase, and/or a NADH:Fd oxidoreductase (NFO), and are thus capable of reoxidizing reduced Fd produced by PFOR. Conversely, *G. thermoglucosidasius* and *B. cereus*, which encode *pdh* but not *pfor*, do not encode enzymes capable of reoxidizing reduced Fd, and thus do not produce H_2_. While the presence of PDH allows for additional NADH production that could be used for ethanol production, *G. thermoglucosidasius* and *B. cereus* end-product profiles suggest that this NADH is preferentially rexodized through lactate production rather than ethanol production. Pyruvate decarboxylase, a homotetrameric enzyme that catalyzes the decarboxylation of pyruvate to acetaldehyde was not encoded by any of the species considered in this study.

Given the requirement of reduced electron carriers for the production of ethanol/H_2_, the oxidative decarboxylation of pyruvate via PDH/PFOR is favorable over PFL for the production of these biofuels. Genome analyses revealed that a number of organisms, including *P. furiosus*, *Ta. pseudethanolicus, Cal. subterraneus* subsp*. tencongensis*, and all *Caldicellulosiruptor* and *Thermotoga* species considered, did not encode PFL. In each of these species, the production of formate has neither been detected nor reported. Unfortunately, many studies do not report formate production, despite the presence of PFL. This may be a consequence of the quantification methods used for volatile fatty acid detection. When formate is not produced, the total oxidation value of 2 CO_2_ per mole glucose (+4), must be balanced with the production of H_2_ and/or ethanol. Thus, the “total molar **r**eduction **v**alues of reduced end-products (H_2_ + ethanol)”, termed *RV*_*EP*_, should be −4, providing that all carbon and electron flux is directed towards end-product formation and not biosynthesis. Indeed, *RV*_*EP*_’s were usually greater than 3.5 in organisms that do not encode *pfl* (*T. maritima*, *Ca. saccharolyticus*), and below 3.5 in those that do encode *pfl* (*C. phytofermentans*, *C. thermocellum*, *G. thermoglucosidasius*, and *B. cereus*; Table 2). In some studies, *RV*_*EP*_’s were low due to a large amount of carbon and electron flux directed towards biosynthesis. In *G. thermoglucosidasius* and *B. cereus RV*_*EP*_’s of H_2_ plus ethanol ranged from 0.4 to 0.8 due to higher reported formate yields. The large differences in formate yields between organisms that encode *pfl* may be due to regulation of *pfl*. In *Escherichia coli*[82,83] and *Streptococcus bovis*[84,85], *pfl* expression has been shown to be negatively regulated by AdhE. Thus presence of *pfl* alone is not a good indicator of formate yields.

#### Genes involved in acetyl-CoA catabolism, acetate production, and ethanol production

The acetyl-CoA/acetate/ethanol node represents the third major branch-point that dictates how carbon and electrons flow towards end-products (Figure 1). Acetyl-CoA may be converted to acetate, with the concomitant production of ATP, either indirectly through an acetyl phosphate intermediate using phosphotransacetylase (*pta*) and acetate kinase (*ack*), or directly via acetate thiokinase *(atk*). Although both reactions produce ATP, the former uses ADP and P_i_ whereas the latter uses AMP and inorganic PP_i_ as substrates for ATP synthesis. As a result, acetate production via *pta* and *ack* is more thermodynamically favorable than via *atk* (△G°’ = −3.9 vs. +6.0 kJ/mol, respectively) which is typically used for acetate assimilation. Of the organisms surveyed, *E. harbinense*, *G. thermodenitrificans*, *C. cellulolyticum*, both *C. thermocellum* strains, and *G. thermoglucosidasius* contain all three genes capable of converting pyruvate to acetate (Table 5). Conversely, *Cal. subterraneus* subsp*. tengcongensis*, *Thermotoga* and *Caldicellulosiruptor* species, *C. phytofermentans*, *Ta. pseudethanolicus*, and *B. cereus* encode only *pta* and *ack*, whereas *P. furiosus* and *Th. kodakaraensis* encode only *atk*.

**Table 5:** Genes encoding proteins involved in end-product synthesis from acetyl-CoA

**Organism**	**gene**					
	***pta***	***ack***	***atk***	***aldH***	***adh***	***adhE***
Standard free energy (G°’)	9.1	−13.0	6.0	17.5	−23.7	−6.2
*Ca. saccharolyticus* DSM 8903	Csac_2041	Csac_2040			Csac_0407	
					Csac_0554	
					Csac_0622	
					Csac_0711	
					Csac_1500	
*Ca. bescii* DSM 6725	Athe_1494	Athe_1493			Athe_0928	
					Athe_0224	
*P. furiosus* DSM 3638			PF1540		PF0075	
			PF1787		PF0608	
*Th. kodakaraensis* KOD1			TK0465		TK1008	
			TK0665		TK1569	
*T. neapolitana* DSM 4359	CTN_0945 CTN_1440	CTN_0411			CTN_0257	
					CTN_0369	
					CTN_0385	
					CTN_0580	
					CTN_1655	
					CTN_1756	
*T. petrophila* RKU-1	Tpet_1042 Tpet_1615	Tpet_0650			Tpet_0007	
					Tpet_0107	
					Tpet_0484	
					Tpet_0508	
					Tpet_0563	
					Tpet_0614	
					Tpet_0813	
*T. maritima* MSB8	TM1130 TM1755	TM0274			TM0111	
					TM0298	
					TM0412	
					TM0436	
					TM0820	
					TM0920	
*Cal. subterraneus* subsp. *tengcongensis* MB4	TTE1482	TTE1481			TTE0313	
					TTE0695	
					TTE0696	
					TTE1591	
*E. harbinense* YUAN-3 T	Ethha_2711	Ethha_2004	Ethha_1333	Ethha_0578	Ethha_0051	Ethha_1385
				Ettha_0635	Ethha_0580	
					Ethha_1164	
					Ethha_2217	
					Ethha_2239	
*C. cellulolyticum* H10	Ccel_2137	Ccel_2136	Ccel_0494 Ccel_1469		Ccel_0894	Ccel_3198
					Ccel_1083	
					Ccel_3337	
*C. phytofermentans* ISDg	Cphy_1326	Cphy_132		Cphy_0958	Cphy_1029	Cphy_3925
				Cphy_1178	Cphy_1421	
				Cphy_1416	Cphy_2463	
				Cphy_1428	Cphy_2463	
				Cphy_2418		
				Cphy_2642		
				Cphy_3041		
*C. thermocellum* ATCC 27405	Cthe_1029	Cthe_1028	Cthe_0551	Cthe_2238	Cthe_0101	Cthe_0423
					Cthe_0394	
					Cthe_2579	
*C. thermocellum* DSM 4150	CtherDRAFT_2741	CtherDRAFT_2742	CtherDRAFT_2349	CtherDRAFT_1042	CtherDRAFT_0189	CtherDRAFT_1096
					CtherDRAFT_0616	
					CtherDRAFT_2833	
*Ta. pseudethanolicus* 39E	Teth39_1296	Teth39_1295			Teth39_0220	Teth39_0206
					Teth39_1597	
					Teth39_1979	
*G. thermoglucosidasius* C56-YS93	Cthe_3862	Geoth_0875	Geoth_0855	Geoth_0268	Geoth_1572	Geoth_3879
			Geoth_0879	Geoth_0652	Geoth_1941	
			Geoth_2349	Geoth_3494	Geoth_0631	
*B. cereus* ATCC 14579	BC5387	BC4637		BC2832	BC0802	BC4365
				BC3555	BC2529	
				BC1285	BC2220	

Abbreviations: *pta*, phosphotransacetylase; *ack*, acetate kinase; *atk*, acetate thiokinase; *aldH*, acetaldehyde dehydrogenase; *adh*, alcohol dehydrogenase; *adhE*; bifunctional acetylaldehyde/alcohol dehydrogenase.

Alternatively, acetyl-CoA may be converted into ethanol, during which 2 NADH (or NADPH) are oxidized, either directly via a fused acetaldehyde/alcohol dehydrogenase encoded by *adhE*, which has been proposed to be the key enzyme responsible for ethanol production [86,87], or indirectly through an acetaldehyde intermediate via acetaldehyde dehydrogenase (*aldH*) and alcohol dehydrogenase (*adh*). While all organisms surveyed encoded multiple class IV Fe-containing ADHs (Table 5), the functions of these ADHs may vary with respect to substrate specificity (aldehyde length and substitution), coenzyme specificity (NADH vs. NADPH), and the catalytic directionality favored (ethanol formation vs. consumption) [10,57-59,72,88-91]. Although there are reports of *in silico* determinations of substrate and cofactor specificity amongst ADHs, in our experience such resolutions are problematic [92,93]. Often times, the gene neighborhoods of identified ADHs were suggestive that the physiological role of many enzymes was not ethanol production. This is evident in *Ca. saccharolyticus*, which does not produce ethanol despite reported NADPH-dependent ADH activity [57].

*P. furiosus*, *Th. kodakaraensis*, and all *Thermotoga* and *Caldicellulosiruptor* species do not encode *adhE* or *aldH*, and therefore produce negligible or no ethanol. Given the absence of ethanol producing pathways in these species, reducing equivalents are disposed of through H_2_ production via H_2_ases and/or lactate production via LDH. Surprisingly, while *Cal. subterraneus* subsp*. tengcongensis* also does not appear to encode *aldH* or *adhE*, NADPH-dependent AldH and both NADH and NADPH-dependent ADH activities, as well as ethanol production, have been reported by Soboh *et al*. [42]. Similarly, *Caldicellulosiruptor obsidiansis*, which does not encode *aldH* or *adhE*, does produce trace levels of ethanol, suggesting that the various encoded ADHs may have broad substrate specificities [94]. Although *C. cellulolyticum* and *Ta. pseudethanolicus* do not encode *aldH*, they do encode *adhE*, and thus are capable of ethanol production. Of the organisms surveyed, only *G. thermoglucosidasius* and *C. cellulolyticum* encoded *aldH* and *adh* but no *adhE*, and produced moderate amounts of ethanol (~0.4 mol per mol hexose). Conversely, a number of organisms (*E. harbinense*, *C. phytofermentans*, both *C. thermocellum* strains, *G. thermoglucosidasius*, and *B. cereus*) encoded *aldH*, *adh*, and *adhE*, all of which produce varying ethanol yields.

#### Hydrogenases

In addition to disposal of reducing equivalents via alcohol and organic acid production, electrons generated during conversion of glucose to acetyl-CoA can be used to produce molecular hydrogen via a suite of [FeFe] and/or [NiFe] H_2_ases. The incredible diversity of H_2_ases has been extensively reviewed by Vignais *et al*. and Calusinska *et al*. [16,95,96]. H_2_ases may be (i) monomeric or multimeric, (ii) can catalyze the reversible production of H_2_ using various electron donors, including reduced Fd and NAD(P)H, or (iii) can act as sensory H_2_ases capable of regulating gene expression [97]. While most H_2_ases can reversibly shuttle electrons between electron carriers and H_2_, they are typically committed to either H_2_-uptake or evolution, depending on reaction thermodynamics and the requirements of the cell *in vivo*[95]. While Fd-dependent H_2_ production remains thermodynamically favorable at physiological concentrations (△G°’ ~ −3.0 kJ mol^-1^), potential production of H_2_ from NAD(P)H (△G°’ = +18.1 kJ mol^-1^) becomes increasingly unfavorable with increasing hydrogen partial pressure [98]. Hence, Fd-dependent H_2_ases are associated with H_2_ evolution, whereas NAD(P)H-dependent H_2_ases are more likely to catalyze H_2_ uptake. Recent characterization of a heterotrimeric “bifurcating” H_2_ase from *Thermotoga maritma* demonstrated that it can simultaneously oxidize reduced Fd and NADH to H_2_ (△G°’ ~ +7.5 kJ mol^-1^), which drives the endergonic production of H_2_ from NADH by coupling it to the exergonic oxidation of reduced Fd [99].

With the exception of *G. thermoglucosidasius* and *B. cereus,* which did not contain putative H_2_ase genes, the genomes of all of the organisms surveyed encode multiple H_2_ases. These H_2_ases were classified based on i) the phylogenetic relationship of H_2_ase large subunits (Additional file 2 and Additional file 3), according to Calusinska *et al*. [16], ii) H_2_ase modular structure, and iii) subunit composition, based on gene neighbourhoods. Encoded [NiFe] H_2_ases fell into 3 major subgroups including: (i) Fd-dependent, H_2_-evolving, membrane-bound H_2_ases (Mbh) and/or energy conserving [NiFe] H_2_ases (Ech) capable of generating sodium/proton motive force (Group 4) [42], (ii) Soluble cofactor-dependent (F_420_ or NAD(P)H), bidirectional, cytoplasmic, heteromultimeric H_2_ases (Group 3), and (iii) H_2_-uptake, membrane bound H_2_ases (Group 1) [96] (Additional file 2). Similarly, encoded [FeFe] H_2_ases fell into 5 major subgroups including: (i) heterotrimeric bifurcating H_2_ases, (ii) dimeric, NAD(P)H-dependent uptake H_2_ases, (iii) monomeric, putatively Fd-dependent H_2_ases, (iv) dimeric sensory H_2_ases containing PAS/PAC sensory domains which may be involved in redox sensing, and (v) monomeric sensory H_2_ases (Additional file 3). These sensory H_2_ases are usually encoded upstream of trimeric bifurcating H_2_ases (Table 6) and are often separated by a histidine/serine kinase suggesting a regulatory relationship between these two enzymes [16].

**Table 6:** **Genes encoding putative hydrogenases, sensory hydrogenases, and NADH:Fd oxidoreductases using ferredoxin, coenzyme F**_**420**_**, and NAD(P)H as electron carriers**

**Organism**	**Hydrogenase and NADH:Fd oxidoreductase classification and corresponding genes**						
	**[NiFe] H**_**2**_**ase**		**[FeFe] H**_**2**_**ase**				**NFO**
	**Fd-dependent *ech *and *mbh***^**G4**^	**F**_**420**_**-dependent**^**G3**^**and other**^**G1**^	**Bifurcating**	**Sensory**^**A**^	**NAD(P)H-dependent**	**Fd-dependent**	***rnf*-type**
Standard free energy (ΔG°’)*	−3.0	11	+7.5**	NA	18.1	18.1	−21.1***
*Ca. bescii* DSM 6725	Athe_1082-Athe_1087		Athe_1297- Athe_1299 ^A1 TR(M3)^	Athe_1292 ^D M2e^			
*Ca. saccharolyticus* DSM 8903	Csac_1534-Csac_1539		Csac_1862- Csac_1864 ^A1 TR(M3)^	Csac_1857 ^D M2e^			
*P. furiosus* DSM 3638	PF1423- PF1436	PF0891- PF0894 ^G3^					
		PF1329- PF1332 ^G3^					
*Th. kodakaraensis* KOD1	TK2080- TK2093	TK2069-TK2072 ^G3^					
*T. neapolitana* DSM 4359			CTN_1067- CTN1069 ^TTH^	CTN_1071- CTN_1072 ^CD(M2f)^	CTN_0485 ^TTH^		CTN_0437-CTN_0442
*T. petrophila* RKU-1			Tpet_1367- Tpet_1369 ^TTH^	Tpet_1371- Tpet_1372 ^CD(M2f)^	Tpet_0723 ^TTH^		Tpet_0675-Tpet_0680
*T. maritima* MSB8			TM1424- TM1426 ^TTH^	TM1420- TM1422 ^CD(M2f)^	TM0201 ^TTH^		TM0244- TM0249
*Cal.subterraneus* subsp. *tengcongensis* MB4	TTE0123- TTE0134		TTE0892- TTE0894 ^A1 TR(M3)^	TTE0887 ^D M2e^			
				TTE0697 ^CD(M2f)^			
*E. harbinense* YUAN-3 T			Ethha_2614- Ethha_2616 ^A8 TR(M3)^	Ethha_0052 ^CD(M2f)^	Ethha_2293 ^A7 D(M3)^	Ethha_0031 ^B2 M2a^	
*C. cellulolyticum* H10	Ccel_1686- Ccel_1691	Ccel_1070-Ccel_1071 ^G1^	Ccel_2303- Ccel_2305 ^A8 TR(M3)^	Ccel_2300- Ccel_2301 ^CD(M2f)^		Ethha_2695 ^B3 M3a^	
	Ccel_3363- Ccel_3371		Ccel_2232- Ccel_2234 ^A1 TR(M3)^				
			Ccel_2467- Ccel_2468 ^A1 TR(M3)^				
*C. phytofermentans* ISDg	Cphy_1730-Cphy_1735		Cphy_0087- Cphy_0089 ^A8 TR(M3)^	Cphy_0092- Cphy_0093 ^CD(M2f)^		Cphy_2056 ^A5 M2c^	Cphy_0211-Cphy_0216
			Cphy_3803- Cphy_3805 ^A1 TR(M3)^	Cphy_3798 ^D M2e^	Cthe_3003-Cthe_3004	Cphy_0090 ^B1 M3a^	
*C. thermocellum* ATCC 27405	Cthe_3013-Cthe_3024		Cthe_0428- Cthe_0430 ^A8 TR(M3)^	Cthe_0425- Cthe_0426 ^CD(M2f)^			Cthe_2430-Cthe_2435
			Cthe_0340- Cthe_0342 ^A1 TR(M3)^	Cthe_0335 ^**D M2e**^			
*C. thermocellum* DSM 4150	CtherDRAFT_2162-CtherDRAFT_2173		CtherDRAFT_1101-CtherDRAFT_1103 ^A8 TR(M3)^	CtherDRAFT_1098-CtherDRAFT_1099 ^CD(M2f)^	Yes^B^		CtherDRAFT_0369-CtherDRAFT_0375
			CtherDRAFT_2978 ^A1 TR(M3)^				
*Ta. pseudethanolicus* 39E				Teth39_0221 ^CD(M2f)^			Teth39_2119-Teth39_2124
			Teth39_1456- Teth39_1458 ^A1 TR(M3)^	Teth39_1463 ^D M2e^			
*G. thermoglucosidasius* C56-YS93							
*B. cereus* ATCC 14579							

^A^Group D M2e hydrogenases are poorly characterized and do not contain a PAS/PAC-sensory domain. However, given their proximity to protein kinases and bifurcating hydrogenases, and their phylogenetic proximity to group C D(M2f) sensory hydrogenases (Additional file 3) we have classified them as sensory hydrogenases.

^B^Verified by microarray and proteomic analysis (*unpublished*).

Characterization of hydrogenase specificity was based metallocenter composition ([NiFe] or [FeFe]), modular structure, subunit composition, and large (catalytic) subunit phylogeny according to Vignais *et al*. and Calusinska *et al*. [16,95,96]. Phylogenetic cluster groupings are indicated in superscript, and corresponding phylogenetic trees are provided in Additional file 1 and Additional file 2. Abbreviations: *H*_*2*_*ase*, hydrogenase; *NFO*, NADH:ferredoxin oxidoreductase; *ech*, energy conserving hydrogenase; *mbh*, membrane bound hydrogenase; *rnf*, *Rhodobacter* nitrogen fixation.

With the exception of *P. furiosus* and *Th. kodakaranesis,* which encode only Fd-dependent and putative F_420_-dependent [NiFe] H_2_ases, all other H_2_ase encoding organisms surveyed are capable of H_2_ase-mediated oxidation/reduction of both Fd and NAD(P)H. This seems fitting given that *P. furiosus* and *Th. kodakaraensis* preferentially catalyze the oxidation of glyceraldedhyde-3-P via GAPFOR rather than GAPDH and PGK, and thus must reoxidize reduced Fd, rather than NADH, during fermentative product synthesis. All other H_2_ase encoding organisms produce NADH during glycolysis and reduced Fd via PFOR. In these organisms, the oxidation of these electron carriers may be carried out using various different types of H_2_ases. All of these species encoded at least a single putative bifurcating H_2_ase (Table 6). The majority of these bifurcating H_2_ases were found downstream dimeric or monomeric sensory [FeFe] H_2_ases that may be involved in their regulation (Table 6). Soboh *et al.* have demonstrated that NADH-dependent H_2_ase activities in *Cal. subterraneus* subsp*. tengcongensis* are affected by H_2_ partial pressures [42] suggesting possible regulation of these H_2_ases via a two-component signal transduction mechanism in response changes in redox levels [16,97]. It is important to note that these NADH-dependent H_2_ase activities may reflect bifurcating H_2_ase activities given that *Cal. subterraneus* subsp*. tengcongensis* encodes only a Fd-dependent and a putative bifurcating H_2_ase, and no NAD(P)H-dependent H_2_ases.

While *Ta. pseudethanolicus* only encodes a bifurcating H_2_ase, all other organisms that encode a bifurcating H_2_ase also encode Fd-dependent H_2_ases. Putative Fd-dependent, [NiFe] Ech/Mbh-type H_2_ases were identified in the genomes of *Cal. subterraneus* subsp. *tengcongensis*, *P. furiosus*, *Th. kodakaraensis*, and all *Caldicellulosiruptor* and *Clostridium* species (Table 6). A pair of putative Fd-dependent [FeFe] H_2_ases were identified in both *E. harbinense* and *C. phytofermentans*. With the exception of *Ta. pseudethanolicus*, *Cal. subterraneus* subsp. *tengcongensis*, and *Caldicellulosiruptor* species, all organisms surveyed containing a bifurcating H_2_ase also appear to be capable of NADH and/or NADPH oxidation using NADH/NADPH-dependent H_2_ases. As with ADHs, however, we could not determine H_2_ase cofactor specificity exclusively using *in silico* sequence analysis, stressing the importance of activity characterization of enzyme substrate specificity. While *C. cellulolyticum* achieves NAD(P)H oxidation using a putative H_2_-uptake [NiFe] H_2_ases, *E. harbinense*, *Thermotoga* species, and *C. thermocellum* ATCC 27405 achieve this using [FeFe] H_2_ases. Although the draft genome of *C. thermocellum* DSM 4150 does not encode an NAD(P)H-dependent H_2_ase, our proteomic and microarray data reveal the presence of Cthe_3003/Cthe_3004 homologues (Rydzak, *unpublished results*).

In addition to H_2_ase-mediated electron transfer between Fd and/or NADH and H_2_, electrons may be transferred directly between Fd and NAD(P)H via an Rnf-like (Rhodobacter nitrogen fixation) NADH:ferredoxin oxidoreductase (NFO), a membrane-bound enzyme complex capable of generating a sodium motive force derived from the energy difference between reduced Fd and NADH. Only *Thermotoga* species, *C. phytofermentans*, *C. thermocellum*, and *Ta. pseudethanolicus* encode putatively identified NFO. Proteomic analysis of *C. thermocellum*, however, revealed low, or no, expression of NFO subunits, suggesting it does not play a major factor in electron exchange between Fd and NADH [100].

While the presence/absence of genes encoding pathways that lead to reduced fermentation products (i.e. formate, lactate, and particularly ethanol) is a major determinant of H_2_ yields, we can make some inferences with respect to H_2_ yields based on the types of H_2_ases encoded. Given the thermodynamic efficiencies of H_2_ production using different cofactors, we can say that Fd-dependent H_2_ases are conducive for H_2_ production while NAD(P)H-dependent H_2_ases are not. However, organisms that do not encode ethanol-producing pathways (i.e. *Caldicellulosiruptor* and *Thermotoga* species) may generate high intracellular NADH:NAD^+^ ratios, making NADH-dependent H_2_ production thermodynamically feasible under physiological conditions. Conversely, in organisms capable of producing both H_2_ and ethanol (*Ethanoligenens*, *Clostridium*, and *Thermoanaerobacter* species), the presence of Fd-dependent H_2_ases appears to be beneficial for H_2_ production. For example, *E. harbinense* and *Clostridium* species, which encode Fd-dependent, as well as bifurcating and NAD(P)H-dependent H_2_ases, produce much higher H_2_ yields when compared to those of *Ta. pseudethanolicus*, which encodes only one bifurcating H_2_ase and no Fd or NAD(P)H-dependent H_2_ases. Interestingly, organisms that do not encode H_2_ases (*G. thermoglucosidasius* and *B. cereus*) produce low ethanol and high lactate (and/or formate yields), suggesting that H_2_ production can help lower NADH:NAD^+^ ratios, and thus reduce flux through LDH.

### Influence of overall genome content on end-product profiles

The presence and absence of genes encoding proteins involved in pyruvate metabolism and end-product synthesis may be used as an indicator of end-product distribution. By comparing genome content to end-product yields, we identified key markers that influence ethanol and H_2_ yields. These include (i) MDH (ii) LDH, (iii) PFL vs. PFOR and/or PDH (iv) Aldh and AdhE, and (v) bifurcating, Fd-dependent, and NAD(P)H dependent H_2_ase.

While it is difficult to elucidate how differences in “malate shunt” genes affect end-product synthesis patterns by comparing reported yields, eliminating MDH has been shown to increase lactate and ethanol production, and decrease acetate production in *C. cellulolyticum*[78]. The elimination of this transhydrogenation pathway may increase NADH:NAD^+^ ratios for reduced end-product synthesis and reduce NADPH:NADP^+^ ratios for biosynthesis. While presence of LDH is not a good predictor of lactate yields, LDH, when activated, diverts reducing equivalents away from H_2_ and ethanol. In contrast to PFL, PFOR and PDH produce additional reducing equivalents (reduced Fd and NADH, respectively), and thus promote reduced end-product synthesis. Organisms that do not encode *pfl* generally produce more ethanol and H_2_ (based on sum redox value) compared to those that do encode *pfl*. Of the organisms surveyed, those that did not encode (or express) both *adhE* and *aldH* produced near-maximal H_2_ yields and little to no ethanol. While the type(s) of encoded H_2_ases appear to have little impact in organisms that do not encode ethanol producing pathways, they do seem to influence reduced end-product yields in those that do. For example, *Ta. pseudethanolicus*, which encodes an *adhE*, NFO, and a single bifurcating H_2_ase, but no discernable Fd or NAD(P)H-dependent H_2_ases, generates low H_2_ and near-optimal ethanol yields. The inability to oxidize reduced Fd via Fd-dependent H_2_ases may elevate reduced Fd levels, which in turn can be used by NFO to produce additional NADH for ethanol synthesis. Interestingly, in the absence of H_2_ases, lactate production was favoured over ethanol production, suggesting that H_2_ production can help lower NADH:NAD^+^ ratios, and thus reduce flux through LDH.

**Figure 2:**
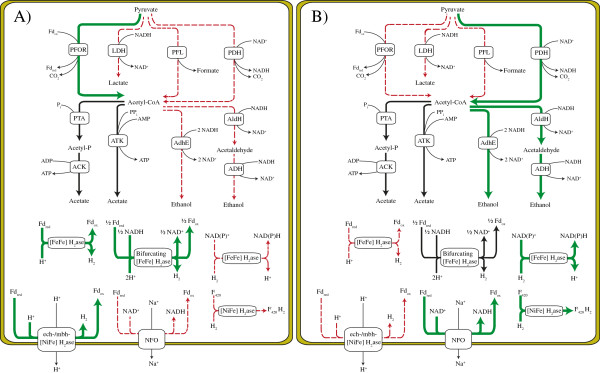
**Differentiation between fermentation pathways that favor (A) hydrogen and (B) ethanol production based on comparative genomics and end-product profiles.** Pathways that favor (green lines), disfavor (broken red lines), and appear to have little impact (black lines) on production of H_2_ or ethanol are indicated. Correlation of reaction thermodynamics and genome content with reported end-product yields suggest that reduction, and subsequent reoxidation, of ferredoxin via PFOR and Fd-dependent (and/or bifurcating) H_2_ases, respectively, support H_2_ production. Alternatively, reduction, of NAD^+^ via PDH (and/or NADH generating uptake H_2_ases) generate NADH conducive for ethanol production. Abbreviations (see figure 1 legend).

Given the impact that MDH, PFL, Aldh, AdhE, and the different H_2_ases have on end-product yields, screening for these biomarkers can streamline ethanol and H_2_ producing potential of sequenced and novel organisms through *in silico* gene mining and the use of universal primers, respectively. Furthermore, understanding how end-product yields are affected by (i) the framework of genes encoding pathways catalyzing pyruvate into end-products, and (ii) thermodynamic efficiencies of these reactions, we can begin to develop informed metabolic engineering strategies for optimization of either ethanol or H_2_ (Figure 2). For example, in order to optimize either ethanol or H_2_, we would recommend elimination of *ldh* and *pfl* in order to allow accumulation of additional reducing equivalents. Given that ethanol and H_2_ compete for reducing equivalents, elimination of one product should direct carbon/and or electron flux towards the other.

For optimization of H_2_ yields (Figure 2A), deletion of *aldH* and *adhE* is likely most effective. Although conversion of pyruvate to acetyl-CoA is more thermodynamically favorable using PDH versus PFOR (△G°’ = −33.4 vs. -19.2 kJ mol^-1^), production of H_2_ from NADH is highly unfavorable compared to the use of reduced Fd (△G°’ = +18.1 vs. -3.0 kJ mol^-1^). This in turn demonstrates that reduction of Fd via PFOR and subsequent H_2_ production via a Fd-dependent H_2_ase (△G°’ = −21.2 kJ mol^-1^) is more favorable than NADH production via PDH and subsequent H_2_ production via NAD(P)H-dependent H_2_ases (△G°’ = −15.3 kJ mol^-1^). Therefore, we propose that conversion of pyruvate to acetyl-CoA via PFOR is favorable for H_2_ production, and *pdh* (and *pfl*) should be deleted. Given that 2 NADH (per glucose) are produced during glycolysis in most anaerobic microorganisms, the presence of a bifurcating H_2_ase, which would simultaneously oxidize the 2 NADH generated during and 2 reduced Fd produced by PFOR, would be required to achieve theoretically maximal H_2_ yields of 4 mol per mol glucose. A Fd-dependent H_2_ase would also be conducive for H_2_ production during times when reducing equivalents generated during glycolysis are redirected towards biosynthetic pathways, resulting in a disproportionate ratio of reduced ferredoxin to NAD(P)H. Alternatively, in organisms such as *P. furiosus* and *Th. kodakaraensis*, which generate high levels of reduced Fd and low levels of NADH, the presence of Fd-dependent H_2_ases, rather than bifurcating H_2_ases, would be more conducive for H_2_ production. In all cases, NFO and NAD(P)H-dependent H_2_ases should be deleted to prevent oxidation of reduced Fd and uptake of H_2_, respectively, which would generate NAD(P)H.

The metabolic engineering strategies employed for optimization of ethanol (Figure 2B) are much different than those used for the production of H_2_. First, *adhE* and/or *aldH* and *adh* genes that encode enzymes with high catalytic efficiencies in the direction of ethanol formation should be heterologously expressed. Given that ethanol production is NAD(P)H dependent, increasing NADH production should be optimized, while Fd reduction should be eliminated. Through deletion of *pfl* and *pfor*, and expression of *pdh*, up to 4 NADH can be generated per glucose, allowing for the theoretical maximum of 2 mol ethanol per mol glucose to be produced. To prevent NADH reoxidation, lactate and H_2_ production should be eliminated by deleting *ldh* and NAD(P)H-dependent H_2_ases. While this strategy is theoretically sound, low AldH/Adh catalytic efficiencies may cause NADH/NAD^+^ ratios to rise so high that they may impede glycolysis. In these situations, the presence of a NFO or NAD(P)H-dependent H_2_ase may intermittently alleviate these high NADH/NAD^+^ ratios through generation of reduced Fd pools or H_2_ production, respectively, albeit it would decrease reducing equivalents for ethanol production.

While some attempts to increase H_2_ and/or ethanol yields through genetic engineering have been successful in a number of lignocellulolytic organisms (reviewed elsewhere; [101]) engineering of strains discussed here has only been marginally successful. Heterologous expression of *Zymomonas mobilis* pyruvate decarboxylase and Adh in *C. cellulolyticum* increased cellulose consumption and biomass production, and decreased lactate production and pyruvate overflow due to a more efficient regulation of carbon and electron flow at the pyruvate branchpoint [102]. However, despite higher levels of total ethanol produced, ethanol yields (per mol hexose consumed) actually decreased when compared to the wild-type strain. Similarly, deletion of PTA in *C. thermocellum* drastically reduced acetate production, but had minimal impact on lactate or ethanol production [103]. This suggests that genome content alone cannot exclusively dictate the extent of end-product yields observed in literature, and thus growth conditions must be optimized in order to moderate regulatory mechanisms that direct carbon and electron flux. This could only be attained through a thorough understanding of regulatory mechanisms that mediate gene and gene-product expression and activity levels under various growth conditions through a combination of genomics, transcriptomics, proteomics, metabolomics, and enzyme characterization.

## Conclusions

Fermentative bacteria offer the potential to convert biomass into renewable biofuels such as H_2_ and ethanol through consolidated bioprocessing. However, these bacteria display highly variable, branched catabolic pathways that divert carbon and electrons towards unwanted end products (i.e. lactate, formate). In order to make fermentative H_2_ and/or ethanol production more economically feasible, biofuel production yields must be increased in lignocellulolytic bacteria capable of consolidated bioprocessing. While the cellulolytic and, to a lesser extent, H_2_ and ethanol producing capabilities of cellulolytic bacteria have been reviewed [8,9,44], a comprehensive comparison between genome content and corresponding end-product distribution patterns has not been reported. While reported end-product yields vary considerably in response to growth conditions, which may influence gene and gene product expression and metabolic flux, we demonstrate that composition of genes encoding pyruvate catabolism and end-product synthesis pathways alone can be used to approximate potential end-product distribution patterns. We have identified a number of genetic biomarkers, including (i) MDH (ii) LDH, (iii) PFL vs. PFOR and/or PDH (iv) Aldh and AdhE, and (V) bifurcating, Fd-dependent, and NAD(P)H dependent H_2_ases, that can be used for streamlining H_2_ and/or ethanol producing capabilities in sequenced and novel isolates. By linking genome content, reaction thermodynamics, and end-product yields, we offer potential targets for optimization of either ethanol or H_2_ yields via metabolic engineering. Deletion of LDH and PFL could potentially increase both H_2_ and ethanol yields. While deletion of ethanol producing pathways (*aldH*, *adh*, *adhE*), increasing flux through PFOR, overexpression of Fd -dependent H_2_ases, and elimination of potential H_2_-uptake (NAD(P)H-dependent) H_2_ases could lead to increased H_2_ production, eliminating H_2_ production and redirecting flux through PDH would be beneficial for ethanol production. Although gene and gene-product expression, functional characterization, and metabolomic flux analysis remains critical in determining pathway utilization, insights regarding how genome content affects end-product yields can be used to direct metabolic engineering strategies and streamline the characterization of novel species with potential industrial applications.

## Abbreviations

ACK: Acetate kinase; ADH: Alcohol dehydrogenase; AdhE: Acetaldehyde/alcohol dehydrogenase (bifunctional); AldH: Aldehyde dehydrogenase; ATK: Acetate thiokinase; Ech: Energy conserving hydrogenase; Fd: Ferredoxin; FDP: Fructose-1,6-bisphosphate; FHL: Formate hydrogen lyase; GAPDH: Glyceraldehyde-3-phosphate dehydrogenase; GAPFOR: Glyceraldehydes-3-phosphate ferredoxin oxidoreductase; H_2_ase: Hydrogenase; IMG: Integrated Microbial Genomes; KO: KEGG Orthology; LDH: Lactate dehydrogenase; MalE: Malic enzyme; Mbh: Membrane-bound hydrogenase; MDH: Malate dehydrogenase; NFO: NADH:ferredoxin oxidoreductase; O/R: (Oxidation/reduction); OAADC: Oxaloacetate decarboxylase; PDH: Pyruvate dehydrogenase; PEP: Phosphoenolpyruvate; PEPCK: Phosphoenolpyruvate carboxykinase; PFK: Phosphofructokinase; PFL: Pyruvate:formate lyase; PFOR: Pyruvate:ferredoxin oxidoreductase; PGK: Phosphoglycerate kinase; PPDK: Pyruvate phosphate dikinase; PPK: Pyruvate kinase; PTA: Phosphotransacetylase; Rnf: Rhodobacter nitrogen fixation; RV_EP_: Total molar reduction values of reduced end-products (H_2_ + ethanol).

## Authors’ contributions

TR and CRC co-authored the manuscript. TV, CRC and TR performed genomic meta-analysis. TR performed end-product comparisons and thermodynamic calculations. CRC performed phylogenetic analysis. RS, NC, and DBL conceived of the study, participated in its design, and helped draft the manuscript. All authors read and approved the final manuscript.

## Supplementary Material

Additional file 1 **Cofactor specificity (ATP or PP**_**i**_**) of phosphofructokinases based on sequence alignments.** Alignments of key residues determining ATP or PP_i_ specificity, as determined by Bapteste *et al*. [74] and Bielen *et al*. [75], were performed using BioEdit v.7.0.9.0. The *P. furiosus* and *Th. kodakarensis* genes are very distinct (different COG and different KO) and are annotated as Archaeal phosphofructokinases. Click here for file

Additional file 2 **Phylogenetic clustering of [NiFe] hydrogenases large (catalytic) subunits.** Catalytic (large) subunits of [NiFe] H_2_ases were identified based upon the modular signatures as described by Calusinska *et al*. [16], Species considered in this manuscript are highlighted and corresponding H_2_ase gene loci are provided. Click here for file

Additional file 3 **Phylogenetic clustering of [FeFe] hydrogenases large (catalytic) subunits.** Catalytic (large) subunits of [FeFe] H_2_ases were identified based upon the modular signatures as described by Calusinska *et al*. [16]. Species considered in this manuscript are highlighted and corresponding H_2_ase gene loci are provided. Click here for file
