# Supplementary material for: Linking genome content to biofuel production yields: a meta-analysis of major catabolic pathways among select H2 and ethanol-producing bacteria
Source: BMC Microbiol. 2012 Dec 18;12:295. doi: 10.1186/1471-2180-12-295 (PMC3561251; doi:10.1186/1471-2180-12-295)
Supplement: Additional file 1 — Cofactor specificity (ATP or PPi) of phosphofructokinases based on sequence alignments. Alignments of key residues determining ATP or PPi specificity, as determined by Bapteste et al. [74] and Bielen et al. [75], were performed using BioEdit v.7.0.9.0. The P. furiosus and Th. kodakarensis genes are very distinct (different COG and different KO) and are annotated as Archaeal phosphofructokinases. [file 1471-2180-12-295-S1.pdf]

6-Phosphofructokinase Genes				
**Organism**	**Locus Tag**	**AA #104**	**AA #124**	**ATP or PPi Dependence**
Bacillus cereus ATCC 14579	BC4600	G	G	ATP
Caldanaerobacter subterraneus subsp. tengcongensis MB4	TTE1816	G	G	ATP
Ethanoligenens harbinense YUAN-3T, DSM 18485	Ethha_1347	G	G	ATP
Geobacillus thermoglucosidasius C56-YS93	Geoth_0897	G	G	ATP
P. furiosus DSM 3638	PF0312	S	E	ATP
	PF1784	L	F	ATP
Thermococcus kodakaraensis KOD1	TK0376	L	F	ATP
	TK1110	S	E	ATP
Thermotoga maritima MSB8	TM0209	G	A	ATP
	TM0289	D	K	PPi
Thermotoga neapolitana DSM 4359	CTN_0395	D	K	PPi
	CTN_0476	G	A	ATP
Thermotoga petrophila RKU-1	Tpet_0623	D	K	PPi
	Tpet_0715	G	A	ATP
Caldicellulosiruptor bescii DSM 6725	Athe_1265	G	G	ATP
	Athe_1824	D	K	PPi
Caldicellulosiruptor saccharolyticus DSM 8903	Csac_1830	G	G	ATP
	Csac_2366	D	K	PPi
Clostridium cellulolyticum H10	Ccel_2223	D	K	PPi
	Ccel_2612	G	G	ATP
Clostridium phytofermentans ISDg	Cphy_0336	G	G	ATP
	Cphy_3345	D	K	PPi
Clostridium thermocellum ATCC 27405	Cthe_0347	D	K	PPi
	Cthe_1261	G	G	ATP
Clostridium thermocellum DSM 4150	CtherDRAFT_1670	G	G	ATP
Thermoanaerobacter pseudethanolicus 39E	Teth39_0494	D	K	PPi
	Teth39_0683	G	G	ATP
