# Supplementary figures and images for: Linking genome content to biofuel production yields: a meta-analysis of major catabolic pathways among select H2 and ethanol-producing bacteria
Source: BMC Microbiol. 2012 Dec 18;12:295. doi: 10.1186/1471-2180-12-295 (PMC3561251; doi:10.1186/1471-2180-12-295)

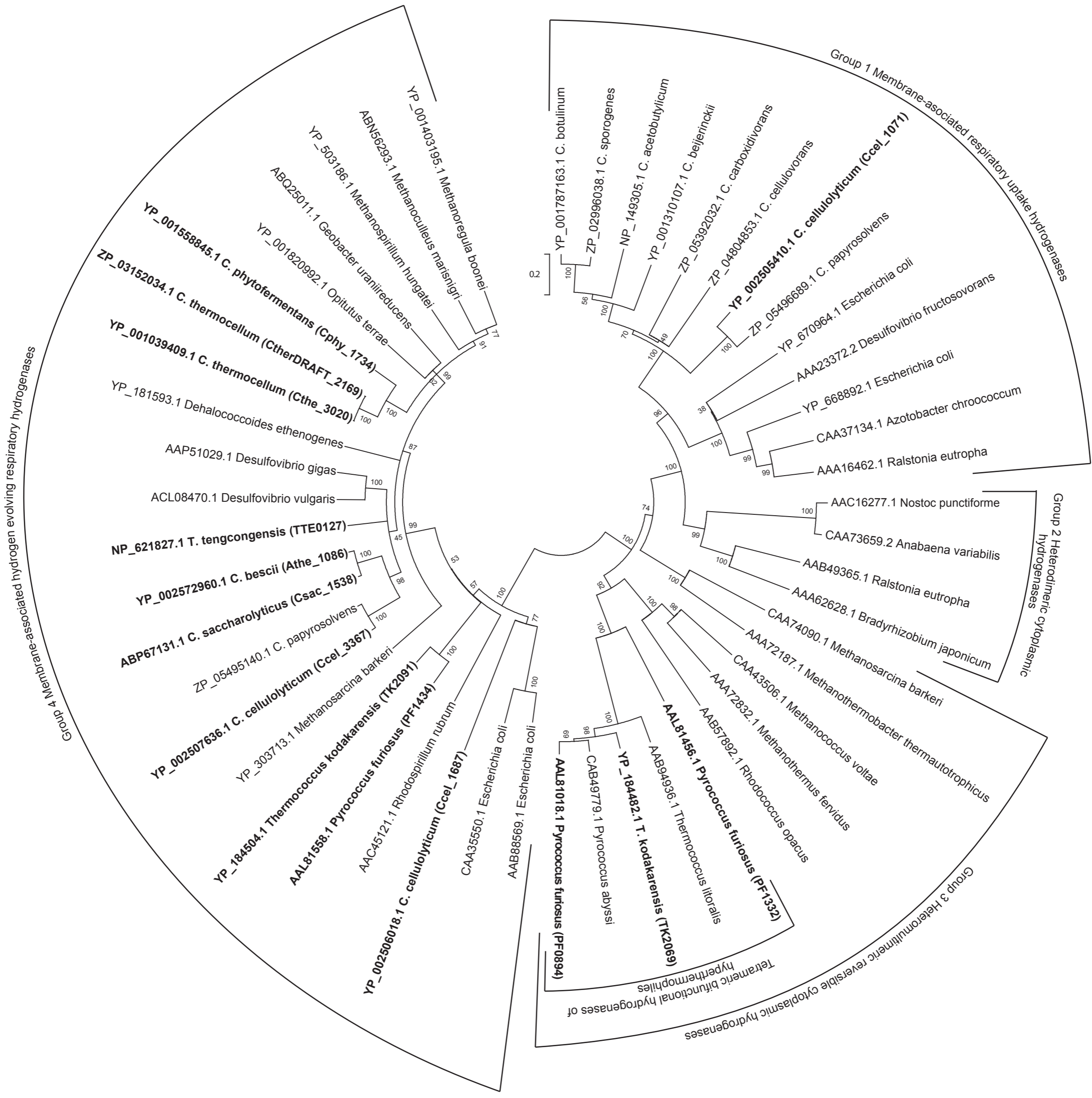

Supplement: Additional file 2 — Phylogenetic clustering of [NiFe] hydrogenases large (catalytic) subunits. Catalytic (large) subunits of [NiFe] H2ases were identified based upon the modular signatures as described by Calusinska et al. [16], Species considered in this manuscript are highlighted and corresponding H2ase gene loci are provided. [file 1471-2180-12-295-S2.pdf]

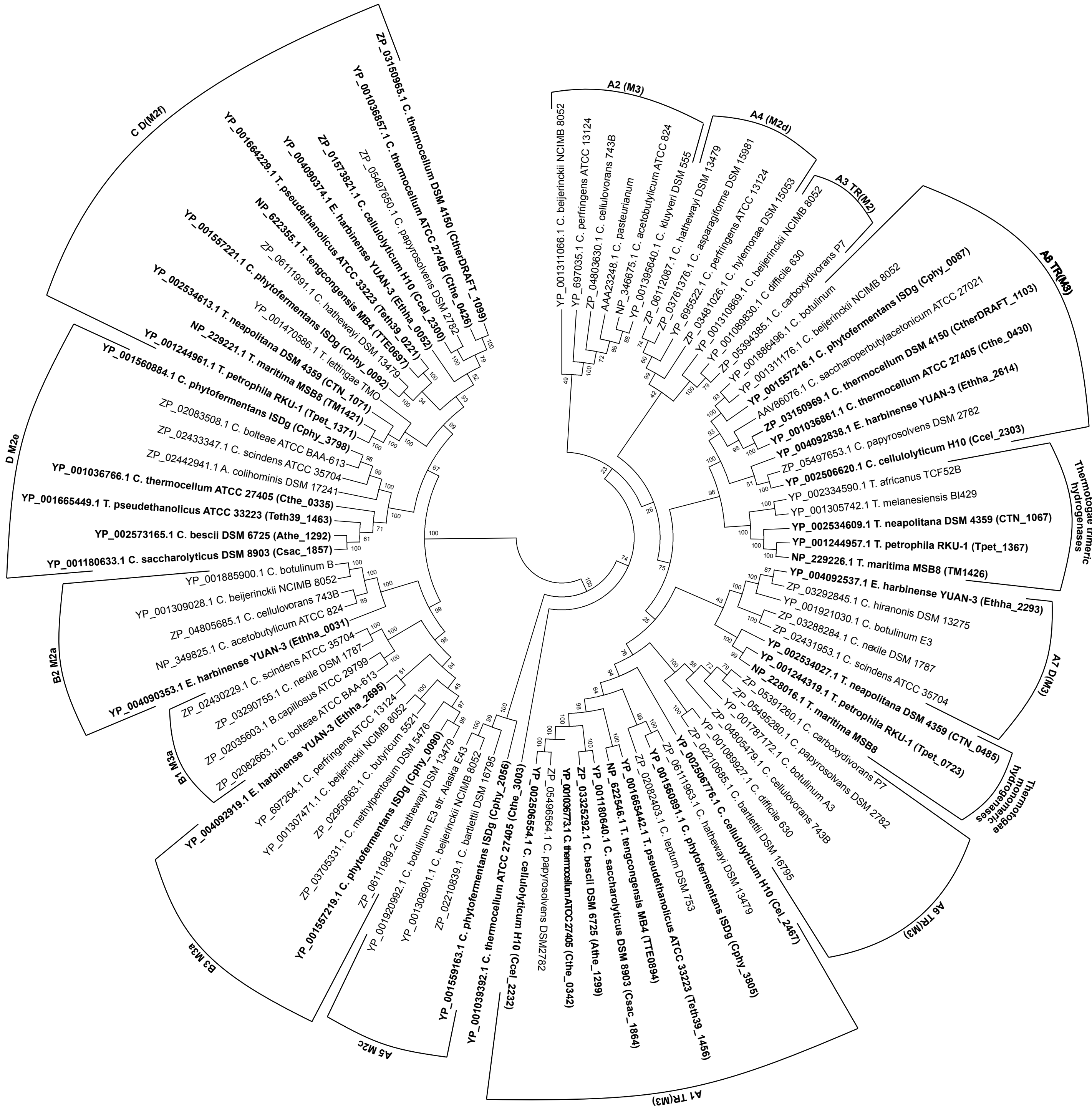

Supplement: Additional file 3 — Phylogenetic clustering of [FeFe] hydrogenases large (catalytic) subunits. Catalytic (large) subunits of [FeFe] H2ases were identified based upon the modular signatures as described by Calusinska et al. [16]. Species considered in this manuscript are highlighted and corresponding H2ase gene loci are provided. [file 1471-2180-12-295-S3.pdf]
